# A phase I trial of panobinostat (LBH589) in patients with metastatic melanoma

**DOI:** 10.1002/cam4.862

**Published:** 2016-10-17

**Authors:** Nageatte Ibrahim, Elizabeth I. Buchbinder, Scott R. Granter, Scott J. Rodig, Anita Giobbie‐Hurder, Carla Becerra, Argyro Tsiaras, Evisa Gjini, David E. Fisher, F. Stephen Hodi

**Affiliations:** ^1^ Department of Medical OncologyDana‐Farber Cancer InstituteBostonMassachusetts; ^2^ Currently at Merck & Co.,KenilworthNew Jersey; ^3^ Department of PathologyBrigham and Women's HospitalBostonMassachusetts; ^4^ Department of Biostatistics & Computational BiologyDana‐Farber Cancer InstituteBostonMassachusetts; ^5^ Department of DermatologyMassachusetts General HospitalBostonMassachusetts

**Keywords:** HDAC, immunotherapy, LBH589, melanoma, MITF, panobinostat

## Abstract

Epigenetic alterations by histone/protein deacetylases (HDACs) are one of the many mechanisms that cancer cells use to alter gene expression and promote growth. HDAC inhibitors have proven to be effective in the treatment of specific malignancies, particularly in combination with other anticancer agents. We conducted a phase I trial of panobinostat in patients with unresectable stage III or IV melanoma. Patients were treated with oral panobinostat at a dose of 30 mg daily on Mondays, Wednesdays, and Fridays (Arm A). Three of the six patients on this dose experienced clinically significant thrombocytopenia requiring dose interruption. Due to this, a second treatment arm was opened and the dose was changed to 30 mg oral panobinostat three times a week every other week (Arm B). Six patients were treated on Arm A and 10 patients were enrolled to Arm B with nine patients treated. In nine patients treated on Arm B, the response rate was 0% (90% confidence interval [CI]: 0–28%) and the disease‐control rate was 22% (90% CI: 4–55%). Among all 15 patients treated, the overall response rate was 0% (90% CI: 0–17%) and the disease‐control rate was 27% (90% CI: 10–51%). There was a high rate of toxicity associated with treatment. Correlative studies suggest the presence of immune modifications after HDAC inhibition. Panobinostat is not active as a single agent in the treatment of melanoma. Further exploration of this agent in combination with other therapies may be warranted.

## Introduction

In the year 2016, it is estimated that 76,380 patients will be diagnosed with melanoma with 10,130 deaths 1. The treatment of melanoma has undergone some dramatic advances over the last several years with the introduction of new immunotherapies and targeted therapies 2, 3, 4, 5, 6, 7, 8. Despite these treatment advances, many patients will still experience progression of their disease. Continued work to understand the modifications which led to melanoma development is essential and may uncover treatments that enhance immunotherapy and targeted therapy.

Epigenetic alterations in chromosome structure play critical roles in the control of gene transcription. Within normal cells, there is a finely balanced system by which histones and other proteins are acetylated and/or phosphorylated. However, in tumor cells, these modifications can become imbalanced leading to repression of tumor suppressor genes or other changes in gene expression 9. Histone deacetylases (HDAC) have been implicated in this process with overexpression in various cancers 10. HDAC inhibitors have been developed as anticancer drugs 11, 12.

HDAC targets both histone and nonhistone proteins within tumor cells 13. Nonhistone proteins regulated by acetylation include *α*‐tubulin, p53, HIF‐1*α,* and HSP90 14. MITF is a transcription factor which plays an essential role in melanoma survival and proliferation and is an amplified oncogene in about 20% of human metastatic melanomas 15. Recent research has indicated an ability of HDAC inhibitor drugs to silence the MITF promoter within all melanoma cell lines 16, 17. The mechanism of silencing appears to be through transcriptional suppression of the *SOX10* gene which negatively affects MITF gene expression. In addition, mouse xenograft experiments using a human melanoma cell line revealed major growth suppression of the melanoma cells when mice were treated with systemic HDAC inhibition 16.

Panobinostat (LBH589) is a potent class I, II, and IV pan‐DAC inhibitor that has shown antitumor activity in preclinical models and cancer patients 18, 19. Based upon the preclinical data in melanoma, the objective of this trial was to obtain early evaluations of LBH589 efficacy in patients with metastatic melanoma. Secondary and correlative objectives were to assess whether LBH589 effectively downregulates MITF in biopsy specimens of treated metastatic melanoma. In addition, we looked at markers of immune regulation to determine if HDAC inhibition influences the immune microenvironment within melanoma tumors.

## Patients and Methods

### Eligibility

This is an open label, phase I trial of LBH589 in patients with metastatic melanoma that is amenable to serial biopsies. Eligible patients had histologically confirmed unresectable stage III or stage IV melanoma, an ECOG performance status of 0–2, normal end‐organ function, and measurable disease defined as at least one lesion 1 cm in greatest dimension. Patients may have received any number of prior therapies as long as these did not include HDAC, DAC, or HSP90 inhibitors and were completed 4 weeks prior to receiving study medication. Patients with known brain metastasis, impaired cardiac function, impaired GI function, or a second malignancy were excluded from the study.

### Study design and treatment

The primary objective of this study was to assess the response rate to panobinostat in patients with metastatic melanoma using RECIST criteria. Secondary objectives were to assess disease‐control rate and time to progression. In addition, correlative studies examined changes in MITF and SOX10 to determine if panobinostat effectively down regulates MITF in biopsy specimens of treated metastatic melanoma patients. Changes in apoptosis markers, phosphorylated ERK (pERK), and AKT were also examined.

A Simon, two‐stage design was utilized with 89% power (target *β *= 0.15) to compare a null response rate of 5% with an alternative response rate of 25%, assuming a one‐sided type I error of 8% (target *α *= 0.1). The target sample size was 22 patients. Nine patients were enrolled in the first stage. If at least one patient exhibited complete or partial response, accrual would continue to the second stage, enrolling 13 additional patients. If three or more among the total of 22 exhibited a response, LBH589 would be considered promising and worthy of further testing.

Six patients were enrolled to the first stage of the trial, Arm A, and were treated with oral panobinostat at a dose of 30 mg daily on Mondays, Wednesdays, and Fridays. This dose was chosen based upon preliminary pharmocologic data which indicated target inhibition at this dose, as well as initial data from the phase I dose escalation trial that this dose could be given safely. The phase I dose escalation trial later determined that 20 mg MWF is the MTD for weekly dosing and 30 mg MWF is the MTD for every‐other‐week dosing. Three of the six patients in Arm A experienced clinically significant thrombocytopenia requiring dose interruption. The decision was made to open Arm B and treat patients with 30 mg oral panobinostat three times (Mondays, Wednesdays, Fridays) a week every other week. The goal was to accrue a total of 22 patients to the every‐other‐week dosing based on the original two‐stage design. The primary efficacy analysis is based on Arm B, with correlative and secondary analysis using the two cohorts combined. A total of 10 patients were enrolled on Arm B with one being replaced due to rapid progression of disease, resulting in nine patients for the analysis of efficacy.

### Assessment of toxicity and response

Safety and tolerability were evaluated according to NCI Common Terminology Criteria for Adverse Events (CTCAE), version 4.0. Treatment was held for grade 3, nonhematologic toxicity and resumed at the same daily dose when this resolved to ≤ grade 1. If the grade 3 toxicity recurred or a grade 4 nonhematologic toxicity occurred, the drug would be held until this resolved to ≤ grade 1 and resumed at a 10 mg lower dose. For hematologic toxicity, treatment was held for grade 4 thrombocytopenia and neutropenia. If the grade 4 thrombocytopenia resolved to ≤ grade 2, then treatment was resumed at a 10 mg lower dose. If neutropenia resolved to ≤ grade 3 within 7 days, treatment was resumed at the same dose, if greater than 7 days treatment was resumed at a 10 mg lower dose.

CT scans of the chest, abdomen, pelvis, and MRI imaging of the brain were performed prior to treatment and restaging scans were obtained every 8 weeks following the initiation of treatment. Tumor responses were determined using RECIST 1.0 (Response Evaluation Criteria in Solid Tumors) criteria.

### Statistical analysis

Demographic and disease characteristics, prior treatment information, and adverse events are summarized descriptively. The overall response rate is defined as the proportion of patients with either complete or partial response (per RECIST criteria) as best response to therapy; disease‐control rate is the proportion of patients with complete or partial response or stable disease. Response and disease‐control rates are presented with 90% exact binomial confidence intervals (CI). Differences in response rates or disease‐control rates by treatment arm were assessed using Fisher's exact test. The distributions of time to progression and overall survival are presented using the method of Kaplan–Meier, with point‐wise, 90% confidence intervals estimated using log(‐log(endpoint)) methodology. Equality of survival curves by arm is assessed using the log‐rank test; however, it should be noted that these tests are of low power due to the small sample sizes. All *P*‐values are two‐sided, with statistical significance defined as *P* < 0.05. There are no corrections for multiple comparisons.

### Correlative analysis

#### Immunohistochemical analysis

Formalin‐fixed, paraffin‐embedded (FFPE) tissue sections of 4‐*μ*m thickness were stained for MART‐1 with anti‐human MART‐1 antibody (clone M2‐7C10) with antigen retrieval for 30 min using Bond Epitope Retrieval 1, primary antibody incubation for 30 min at 1:100. Staining for Sox10 was done with a polyclonal anti‐human Sox10 antibody with antigen retrieval for 30 min using Bond Epitope Retrieval 1, primary antibody incubation for 30 min at 1:50. Staining for pERK was performed with anti‐human pERK [(Erk1/2) (Thr202/Tyr204)] with antigen retrieval for 30 min using Bond Epitope Retrieval 1, primary antibody incubation for 30 min at 1:150. TUNEL staining was performed using the Millipore (Darmstadt, Germany) ApopTag Peroxidase In Situ kit on the Bond III.

## Results

### Patients and treatment

Trial enrollment began on May 5, 2010 and six patients were enrolled to the first stage of the trial (Arm A) and treated with oral panobinostat at a dose of 30 mg daily on Mondays, Wednesdays, and Fridays. However, three of the six patients experienced clinically significant thrombocytopenia requiring dose interruption and the dosing was changed to 30 mg oral panobinostat three times a week every other week (Arm B). A total of 10 patients were enrolled following the dose change with one being replaced due to rapid progression of disease resulting in nine patients for the analysis of efficacy. Enrollment completed on December 10, 2012.

The baseline patient characteristics for all 16 of the patients enrolled are shown in Table 1. Overall, the patients were Caucasian, non‐Hispanic, and predominantly male. The median age was 63 and the majority had an ECOG status of 0 and stage IV disease. All patients had received prior cancer‐directed surgery with three patients receiving prior chemotherapy and radiation (19%), three patients having radiation therapy (19%), and seven patients receiving chemotherapy (44%). None of the patients had known brain metastasis at the time of study enrollment.

**Table 1:** Baseline demographic characteristics

	Arm A (*n* = 6)	Arm B (*n* = 10)	Total (*n* = 16) (%)
Male	3	4	7 (44)
Female	3	6	9 (56)
Stage III	0	2	2 (13)
Stage IV	6	8	14 (88)
ECOG 0	5	5	10 (63)
ECOG 1	1	4	5 (31)
Unavailable	0	1	1 (6)
Histology			
Cutaneous	2	4	6 (38)
Mucosal	1	0	1 (6)
Acral lentiginous	0	1	1 (6)
Ocular	0	1	1 (6)
Unknown primary	3	4	7 (44)
Prior therapy			
Radiation	3	3	6 (38)
Chemotherapy	2	8	10 (63)
Unavailable	1	1	2 (13)

The median number of months on treatment was 1.9 (range: 0.2–21.4) and the median number of months on study was 6.7 (range: 1.7–25.2). Median follow‐up was 6.8 months (range: 1.7–25.2 months). All patients were off‐study at the time of the data retrieval and all patients developed progressive disease. Three patients (20%) were alive at the time of the data analysis.

### Efficacy

The primary analysis of efficacy was based upon nine patients treated on Arm B. The response rate was 0% (90% exact CI: 0–28%) and the disease‐control rate was 22% (90% exact CI: 4–55%). Since there were no patients with response (CR or PR) among the first nine patients enrolled in Arm B, the continuation criteria for the first stage of the Simon two‐stage design were not satisfied, and the study was stopped.

**Figure 1:**
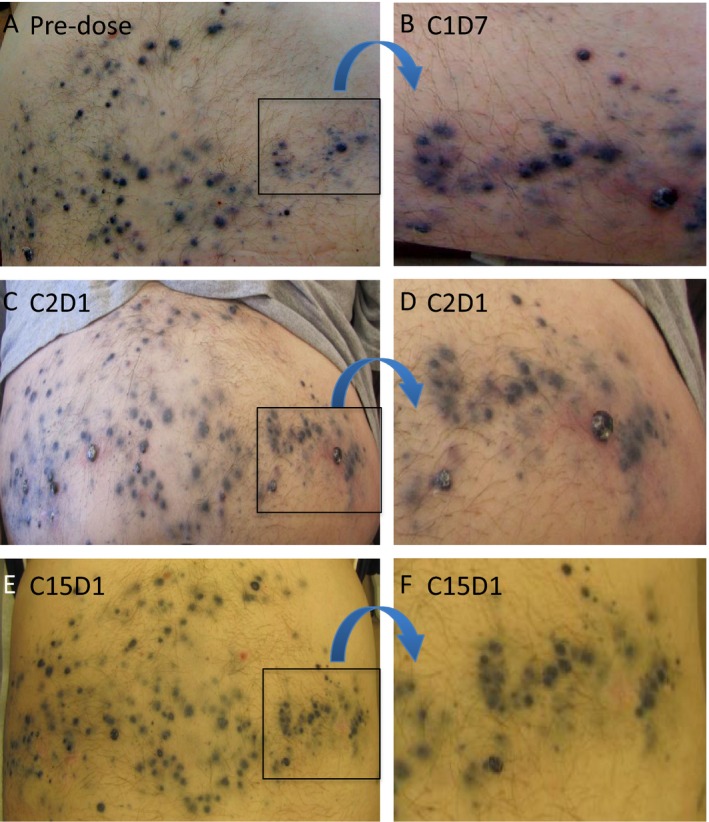
Subject in Arm A who received a total of 15 cycles of panobinostat. His abdomen was heavily involved with in‐transit metastases; (A) predose image. At C1D7, he had inflammation surrounding the in‐transit metastases and with necrosis of some of the larger nodules (B). At the end of cycle 1, there was evidence of hypopigmentation of the in‐transit metastases as well as flattening of many of the raised nodules with a decrease in surrounding inflammation (C) with enlarged area in (D). After 15 cycles of panobinostat (E), all of the in‐transit metastases were flat and markedly hypopigmented. Note the improvement in the highlighted area (F) that was very inflamed and necrotic at C1D7 (B). There were no new in‐transit metastases that developed while the subject was on study. The subject eventually had disease progression in an axillary lymph node and came off‐study.

In an aggregated analysis based on all 15 patients, no patient achieved a partial or complete response. Four patients (two in Arm A and two in Arm B) achieved stable disease. One patient in Arm A achieved SD for 16 months on study. His disease included in‐transit disease across his abdomen and involvement of an axillary lymph node. There was evidence of regression around the in‐transit metastases (hypopigmentation and flattening of raised lesions) as well as necrosis (see images presented in Fig. 1). The overall response rate was 0% (90% exact CI: 0–17%) and a disease‐control rate of 27% (90% exact CI: 10–51%). Patient response data are shown in Table 2.

**Table 2:** Patient response and status

	Arm A (*n* = 6)	Arm B (*n* = 9)	Total (*n* = 15)
Best overall response			
Stable disease	2	2	4 (27%)
Progressive disease	4	7	11 (73%)
Survival status			
Alive	1	2	3 (20%)
Dead	5	7	12 (80%)
Months on treatment			
Mean	5.3	2.4	3.6
S.D.	8.0	2.7	5.4
Median	2.1	1.8	1.9
Months on study			
Mean	10.5	6.4	8.1
S.D.	10.2	3.6	7.0
Median	6.2	6.7	6.7

**Figure 2:**
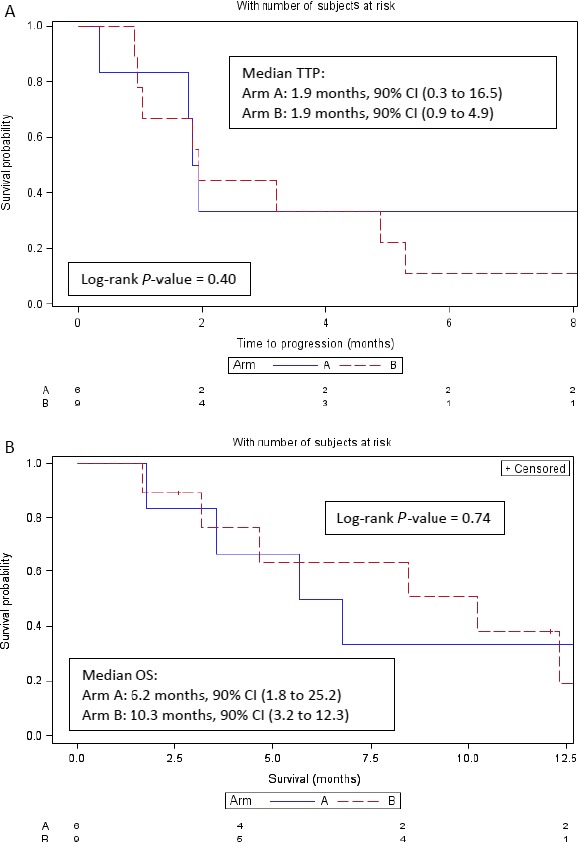
Time to progression and overall survival—by Arm.

For Arms A and B together (*N* = 15), median time to progression was 1.9 months (90% CI: 1.1–4.9 months). There was no evidence of a difference in time to progression by treatment arm (log‐rank *P* = 0.40). A total of three patients (20%) from Arms A and B were alive at the time of the data retrieval (3/31/14). Kaplan–Meier estimates of time to progression and overall survival are shown by treatment arm in Figure 2. Median overall survival was 8.4 months (90% CI: 3.6–12.3 months). Median survival in Arm A was 6.2 months (90% CI: 1.8–25.2 months) and was 10.3 months in Arm B (90% CI: 3.2–12.3 months). There was no difference in survival by arm (log‐rank *P* = 0.74).

### Toxicity

Toxicities and adverse events were classified according to CTCAE Version 4.0 (NIH, Bethesda, MD, USA). Toxicity data were collected for all enrolled patients for the entire follow‐up period. All 16 patients reported adverse events during the trial with a median of 9.5 unique types of AEs per patient (range: 1–29). A summary of the number of AEs graded 2 or higher considered to be possibly, probably, or definitely related to study therapy are presented in Table 3. There were no grade 4 or 5 drug‐related toxicities. Median time to onset of any toxicity was 4 days with a median time of 5 days for toxicities rated as possibly, probably, or definitely related to treatment. Treatment was held due to toxicity in six of the 13 patients (37.5%): three in Arm A (50%) and three in Arm B (30%). None of the patients came off‐study due to toxicity; all had treatment discontinued due to progressive disease.

**Table 3:** Adverse events grade 2 or higher reported as possibly, probably, or definitely related to treatment

Description	Grade		
	2	3	4
Hematologic toxicity			
Thrombocytopenia	3	3	
Anemia	2		
Neutropenia	3		
Lymphocytopenia		3	
Gastrointestinal toxicity			
LFT elevation	1	1	
Diarrhea	1		
Nausea	5		
Vomiting	2		
Musculoskeletal toxicity			
Back pain	1		
Fatigue	4		
Muscle weakness	1		
Renal toxicity/electrolyte abnormalities			
Creatinine increase	1		
Hypophosphatemia	1	1	
Hypokalemia		1	
Infectious disease			
Skin infection	1		
Cardiac toxicity			
Flattened R wave	1		

One death was reported on study which was due to progression of disease in a patient who was off‐study drug at the time of death. The majority of severe toxicities observed were hematologic toxicities including thrombocytopenia, anemia, neutropenia, and lymphocytopenia. There was also a high rate of nausea, vomiting, and fatigue reported on the study treatment as presented in Table 3.

### Correlative analysis

**Figure 3:**
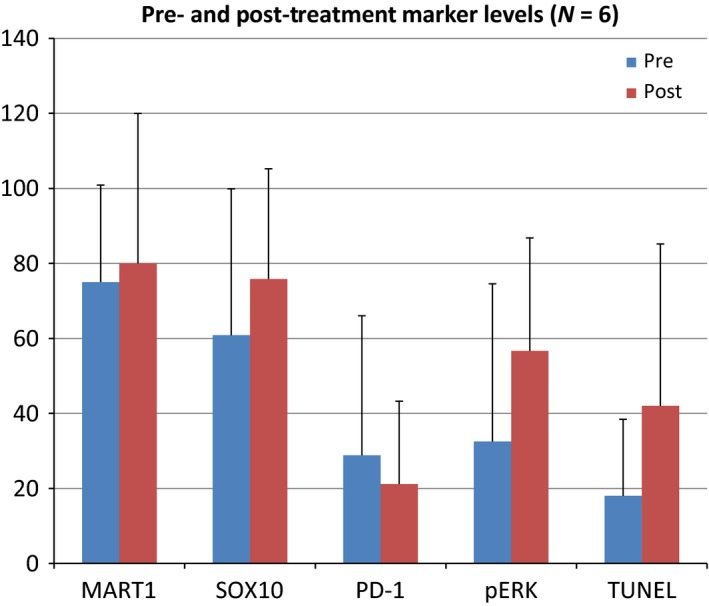
Pre‐ and Posttreatment marker levels (*N* = 6). Patient's tumors were stained by IHC pre‐ and posttreatment with panobinostat. None of the changes pre‐ and posttreatment were statistically significant. *P* ‐value between time points for each marker MART‐1 *P* = 0.80, SOX10 *P* = 0.50, PD‐1 0.39, pERK *P* = 0.16, TUNEL *P* = 0.23. pERK, phosphorylated ERK.

**Figure 4:**
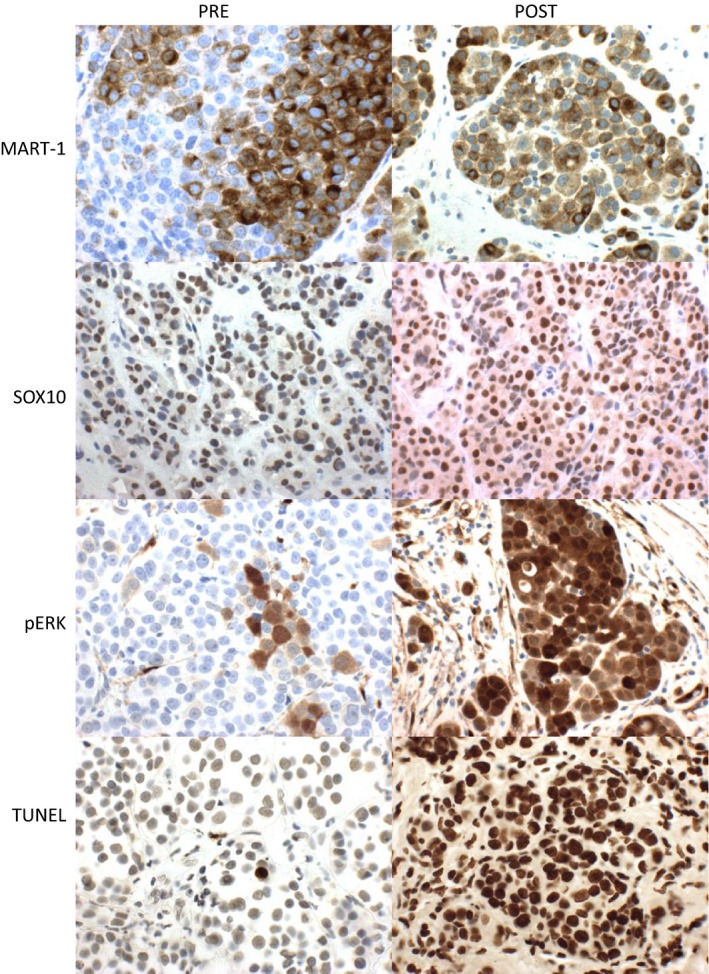
Representative Pre‐ and postimmunohistochemistry staining for markers of MITF.

Biopsies were obtained pre‐ and posttreatment with panobinostat and stained for Mart‐1, Sox‐10, and pERK to evaluate if panobinostat downregulates MITF. Six paired patient tumor biopsies were examined for pERK, PD1, TUNNEL, SOX10, and MART1. Staining for pERK was performed given the interaction between MITF and the MAPK pathway as evidenced by lack of response to BRAF inhibition in MITF‐overexpressing cell lines. Results of this staining are presented in Figure 3. There were no statistically significant differences observed between the different marker levels. This lack of statistical significance was likely due to small sample sizes and the large variability in the measurements. Representative images of the staining are presented in Figure 4.

**Figure 5:**
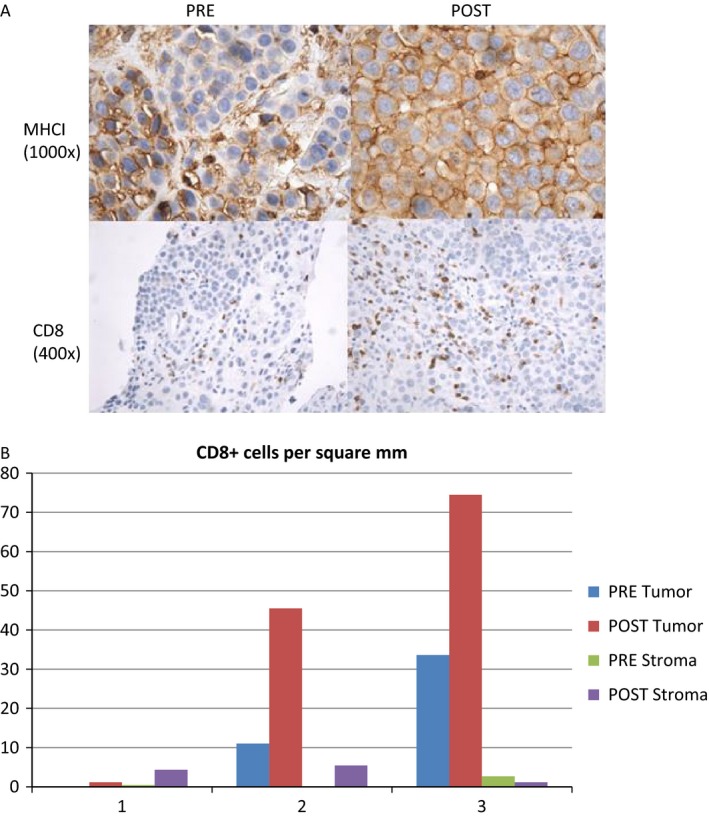
(A) Representative pre‐and postimmunohistochemistry staining for immunologic markers. (B) Number of CD8 + cells per square mm pre‐and posttreatment with panobinostat in the tumor and surrounding stroma.

In addition, staining was performed on three available paired tumor biopsies to look at markers of immune function including PD‐1, PD‐L1, MHCI, MHCII, and CD8 positive. There was no change observed in PD‐1 or PD‐L1 staining between the pre‐ and post specimens. In addition, there was no clear difference in MHCII staining. There did appear to be an increase in MHCI staining between the pre‐ and post biopsies. In addition, the post specimens had an increase in the infiltrating CD8 + T cells when compared to the pretreatment specimens. Representative images of the staining are presented in Figure 5.

## Discussion

Epigenetic modification plays a role in melanoma development which suggested that HDAC inhibition would be potentially promising therapeutic in this disease. Unfortunately, as a single agent, panobinostat is not effective in the treatment of advanced melanoma and was difficult to tolerate due to high rates of thrombocytopenia.

In this trial, two cohorts of patients were enrolled with different dosing schedules. Arm A utilized weekly dosing and Arm B had every‐other‐week dosing. Per protocol, the primary efficacy analysis of response and disease‐control rates was based on Arm B, although aggregated response rates are also reported. In Arm B, the response rate was 0% and the disease‐control rate was 22%. Since there were no patients with a response (CR or PR) among the first nine patients, the study was stopped for lack of efficacy at the futility analysis. For the study cohort overall (*N* = 15), the response rate was 0% and the disease‐control rate was 27%.

Panobinostat was poorly tolerated overall with the need to reduce the dosing on the study from weekly to every other week. Patients experienced hematologic toxicities as well as nausea, vomiting, and fatigue. Toxicity may have limited the ability of patients to receive sufficient therapy to suppress MITF and achieve a response.

Despite prior in vitro and xenograft experiments showing growth suppression of melanoma and suppression of MITF with HDAC inhibition, this was not observed in patients. Tissue obtained from patients before and after treatment was stained to determine if panobinostat therapy led to alterations in MART‐1, SOX10, and pERK as markers of MITF alteration. Staining for MART‐1 and SOX10 did not show a significant change with therapy. However, these correlative studies were limited by the small sample size and large variability among measurements. In addition, the high toxicity of the therapy lead to reduced dosing at which detection of alterations in MITF may have been more challenging.

In this study, there was some nonsignificant increase in pERK staining observed in the posttreatment samples. This could be related to suppression of MITF leading to increased MAPK signaling. However, there are numerous proteins that interact with MAPK pathway that could be affected by epigenetic modification. HDACs have been implicated as a mechanism of resistance to BRAF inhibitors through development of aberrant apoptotic pathways 20. HDAC inhibition in combination with BRAF inhibition has displayed synergy in preclinical testing 17, 21, 22. The increase in pERK signaling seen in this study further supports a future combination of HDAC inhibition with agents targeting the MAPK pathway.

All tumors expressed PDL‐1 at baseline and treatment with panobinostat alone was not effective in decreasing PDL‐1 tumor expression. This indicates that combination strategies may be more successful and mediate an antitumor response.

In the treatment of myeloma, panobinostat has proven most effective in combination therapy and is given with bortezomib and prednisone 19. It is possible that the limited activity which we are seeing in melanoma reflects the need for better combination strategies. Bortezomib has not proven to be particularly effective in melanoma and is not a candidate for combination as witnessed by the negative phase I trial combining sorafenib and bortezomib in this disease 23. A combination of decitabine, panobinostat, and temozolomide in metastatic melanoma was well tolerated in a phase I clinical trial with some evidence of activity with a 75% disease‐control rate 24.

Immunotherapy has revolutionized the treatment of melanoma and is yielding exciting results in the treatment of other malignancies as well 2, 25, 26, 27, 28. However, only a subset of patients respond to this therapy and there remains a need to increase the efficacy of immunotherapy through combinations. One potential combination would be with agents that modify the epigenetic environment within tumor cells, immune cells, and the surrounding stroma. In this study, an increase in MHCI staining and CD8 + T‐cell infiltration was observed following treatment with panobinostat in a subset of the patients. This finding is hypothesis‐generating and supports the rationale for combination of HDAC inhibitors with immune checkpoint blockade in the treatment of melanoma.

The relationship between HDAC inhibitors and the immune system is very complex with both immune‐suppressive and immune‐stimulatory effects observed. There have been reports that HDAC inhibitors upregulate genes associated with antigen presentation including MHCI and MHCII 29, 30, 31. This leads to increased recognition of the tumor by NK and T cells. In this study, we observed an increase in MHCI staining following treatment with panobinostat.

One issue that goes against the use of HDAC inhibition in combination with immunotherapy is the finding that HDAC inhibition promotes expansion and function of T‐regulatory cells 32, 33. In addition, HDAC inhibitors are reported to have inhibitory effects on CD4 + T‐cell viability and function 34. These findings suggest that appropriate sequencing and selection of agents will be essential to any combination of HDAC inhibition and immunotherapy.

In this study, we showed that single agent panobinostat is not effective in the treatment of melanoma. However, the correlative work performed in this study suggests a role for future exploration of HDAC inhibition in combination with targeted therapies and immunotherapy.

## Conflicts of Interest

Novartis provides clinical trial support to the authors. N Ibrahim is employed by Merck & Co.
